# Effect of Gas Exchange Rate, Vessel Type, Planting Density, and Genotype on Growth, Photosynthetic Activity, and Ion Uptake of In Vitro Potato Plants

**DOI:** 10.3390/plants13192830

**Published:** 2024-10-09

**Authors:** Rainer Vollmer, Janeth Espirilla, Ana Espinoza, Rosalva Villagaray, Mario Castro, Sandra Pineda, Juan Carlos Sánchez, Alexandre F. S. Mello, Vania C. R. Azevedo

**Affiliations:** International Potato Centre (CIP), Av. La Molina, La Molina, Lima 15023, Peru

**Keywords:** *Solanum*, leaf area, stem length, propagation, quality aspects

## Abstract

The growth of high-quality in vitro potato plants (*Solanum stenotomum* subsp. *stenotomum*, *Solanum stenotomum* subsp. *goniocalyx*, and *Solanum tuberosum* subsp. *andigena*) is affected by multiple biological, operational, and environmental factors. Research on in vitro culture is frequently focused on the species, explant, composition of the culture medium, and incubation conditions, but only limited information is available on the effect of the gas exchange rate and volume of in vitro culture vessels under variable planting densities. In the present study, these factors were evaluated with a set of seven diverse potato landraces. The results were compared to the plants’ responses in routinely used in vitro culture vessels, i.e., 13 × 100 mm and 25 × 150 mm test tubes, and GA7^®^ magenta vessels. In vitro potato plants grown in plastic vessels equipped with a HEPA filter delivering a high gas exchange rate developed thicker stems (0.95 mm), a higher total average leaf area (2.51 cm^2^), increased chlorophyll content in leaves (32.2 ppm), and lower moisture content in their tissues (90.1%) compared to filter systems with lower gas exchange rates. A high planting density of 10 × 10 plants per vessel (360 and 870 mL) negatively affected the average stem width and root length but increased the plant height (3.4 cm). High fluctuations of ion-uptake of NO_3_^−^, Ca^++^, K^+^, and Na^+^ were observed between genotypes, with some accessions having a 4.6-times higher Ca^++^-ion concentration in their tissues (190–234 ppm). The in vitro plants developed more robust stems, longer roots, and larger leaves within in vitro culture vessels equipped with a HEPA filter (high gas exchange rate) compared to the control vessels, in contrast to the chlorophyll content in leaves, which was higher in plants grown in narrow test tubes. Depending on the purpose of the subculture of in vitro plants, their growth and development can be molded using different gas exchange rates, planting densities, and vessel volumes.

## 1. Introduction

Potato is considered the third most important crop worldwide, with a global production of 374 million metric tons, cultivated on an area of about 19 million hectares [1]. The cultivated form of potato covers seven species and four ploidy levels [2], including more than 4500 landraces. Potato is propagated vegetatively and grows from sea level up to 4600 m.a.s.l. at nearly any latitude. It provides food security to millions of people as a low-fat source of carbohydrates and is rich in vitamin C, iron, zinc, and potassium [1,2]. Since potato is a clonally propagated crop, large-scale multiplication and distribution within seed production systems are accomplished through the application of in vitro culture techniques. The in vitro plants are grown under controlled environmental conditions on solid or liquid culture media within special glass or plastic vessels. In this manner, a high number of plants can be produced in a short time period, and disease-free potato plants (e.g., those free of viruses, bacteria, and fungi) are maintained phytosanitarily clean over time, which are two of the most important criteria for high-quality seed systems [3]. The first reports of in vitro potato culture date from more than 70 years ago [4]. Over time, multiple applications of in vitro culture of potato plants have been developed, like meristem and shoot culture, cold storage, callus induction, protoplast production, and the induction of somaclonal variation [5,6,7]. It soon became clear that successful propagation of in vitro potato plants using single nodes as explants does not require additional plant growth regulators (PGRs) and can be performed on standard Murashige Skoog medium (MS) [8,9]. In contrast, the addition of PGRs like 6-Benzylaminopurine (BAP) and/or 1-Naphthaleneacetic acid (NAA) produced callus formation prior to shoot growth, involving the potential risk of causing unwanted somaclonal changes during germplasm conservation [10]. Although in vitro potato plants can be grown on solid and liquid culture media, a higher shoot length, number of nodes, and shoot and root dry weight were observed on liquid culture medium [11]. However, for conservation and distribution purposes, it is preferred to use a solid culture medium instead of a liquid culture medium. It was possible to prolong the period between subsequent sub-culture cycles of in vitro potato plants by adding polyol osmolytes (2–4% of sorbitol) and sucrose (2%) to the culture medium [12]. At CIP, in vitro potato plants are propagated on standard solid MS medium (without PGRs) and distributed with a plant age of 3–5 weeks [13]. In addition to the culture medium’s composition, the growth and development of in vitro potato plants are influenced by environmental factors. In vitro potato plants showed the best growth with a photoperiod of 16 h of day length and a temperature of 20 °C [8]. Under a light intensity of 100 μmol m^−2^ s^−1^ (provided by light-emitting diodes, LEDs), in vitro potato plants developed thicker stems, a higher chlorophyll content, and increased fresh and dry matter compared to a light intensity of 50 μmol m^−2^ s^−1^, whereas shoots were significantly longer with the lower light intensity of 50 μmol m^−2^ s^−1^ [14]. Beside the above-described factors, other less studied physical conditions can have a major impact on the growth and development of in vitro plants, like the culture vessel’s gas exchange rate, volume, material, and closure type [15]. Also, the number of plants cultivated per vessel, or planting density, is an important component to be considered, as it permits the modulation of morphological and quality aspects of the plants, depending on the objective of the subculture or conservation activity (i.e., short-term in vitro conservation in active collections and nurseries and mid-term in vitro conservation under cold-storage conditions).

The gas exchange rate of culture vessels is calculated by dividing the hourly volumetric ventilation rate by the gas volume of the vessel. The gas exchange rate varies depending on the type of filter the vessel is provided with, sealing (without filter), vessel volume, type of tissue, and environmental conditions. The gas exchange rate is particularly related to the flux of CO_2_, O_2_, ethylene, and water vapor in and/or out of the vessel [16]. It was shown that larger filter diameters of in vitro culture vessels of 2.0 and 2.5 cm (higher gas exchange rate) increased plant growth, water content, and leaf number in four potato cultivars (*Solanum tuberosum*) compared to smaller filters of 1.0 and 1.5 cm [17]. Similarly, in vitro potato plantlets (cv. ‘Bintje’) cultivated in non-hermetically sealed tubes (higher gas exchange rate) developed a 1.7-fold higher maximum photosynthetic rate than plants grown in hermetically sealed tubes [18]. Also, the contents of chlorophyll a and b and β-carotene were significantly higher in leaves when in vitro tobacco plants (*Nicotiana tabacum* L.) were grown in Magenta GA-7 vessels equipped with microporous vents instead of tightly closed glass vessels. In vented vessels, the leaves transpired less, had a lower stomatal conductance, and had a higher net photosynthetic rate [19]. In contrast, a more than 20-fold higher ethylene accumulation was observed when in vitro potato plants (cv. ‘Cara’) were grown in sealed vessels, compared to poly-propylene membrane-equipped vessels (diffuse ventilation) or vessels mounted with a forced-ventilation apparatus (5 cm^3^.min^−1^). The oxygen concentration in the headspace of the vessels with diffuse (18%) and forced ventilation (20%) was significantly higher than in sealed vessels (15%). Consequently, leaf fresh mass, leaf area, stem height, and root length were also significantly higher under diffuse and forced ventilation conditions [20]. Finally, the assessment of various filter and sealing types showed vessels sealed with a double layer of tape had a significantly lower gas exchange rate compared to vessels sealed by a single layer, provided with a cotton filter, or a combination of both [21]. On the molecular level, a higher gas exchange rate facilitates the entry and exit of CO_2_ molecules from the vessel. Generally, in vitro plants are grown under heterotrophic conditions, under which the plants avoid photosynthesis as long as there is a suitable carbohydrate source available in the medium (usually sucrose) [7]. Alternatively, in vitro plants can be grown in a photoautotrophic system on a carbohydrate-free culture medium, demanding a high CO_2_ concentration and light intensity in the vessel. Under these conditions, plant growth and the accumulation of carbohydrates are fully dependent upon photosynthesis and inorganic nutrient uptake [22]. On the other hand, it was reported that CO_2_ enrichment also favored shoot multiplication, shoot elongation, and biomass production in sucrose-supplemented medium (heterotrophic conditions) [23]. Moreover, the removal of CO_2_ decreased the growth of in vitro *Arabidopsis* seedlings by 50% in the presence of sucrose in the medium, while the addition of sucrose as a replacement for photosynthesis resulted in only a partial recovery of growth [24]. Based on this evidence, one could assume that CO_2_ is favorable and required (at least at a minimum level) for growing in vitro plants under heterotrophic conditions. Further, a higher gas exchange rate affected the leaf anatomy. Under a high gas exchange rate, a reduction of stomatal cells was observed in the leaves of *Vernonia condensata*, along with an increase in the photosynthetic rate and the concentration of hexoses and starch in the leaves [25]. Also, the ion content in plant tissues was affected by the availability of CO_2_ (gas exchange). During in vitro culture of *Theobroma cacao*, potassium (K) content in plant tissues was proportional to shoot growth and greatest under enriched CO_2_ conditions, while calcium content (Ca) was not associated with shoot growth [26]. 

Beside the gas exchange rate, the planting density within the vessel plays an important role for growth and development of potato in vitro plants. Planting density can be defined as the number of in vitro plants located in a certain area of culture media, resulting in variable distances between adjacent plants. Increasing the planting density from one to five in vitro potato plants (cv. ‘Desiree’) per test tube did not significantly affect shoot length and number of roots per plant after four weeks of growth. In contrast, plants grown under the highest planting density (five plants/tube), developed a significantly higher number of nodes per plant, compared to lower densities of one to three plants per tube [27]. In a similar study, a high planting density of five plants per test tube (vs. one, two, three or four plants per tube) did not significantly affect shoot length, number of roots, or number of nodes of in vitro potato plants (cvs. ‘Kufri Jyoti’ and ‘Kufri Chandramukhi’) after four weeks of culture. No significant differences were observed for the interaction of genotype x planting density for any of the assessed growth variables [28]. Similarly, the growth of blueberry (varieties ‘Brigitta’ and ‘Legacy’) under in vitro conditions was significantly affected by the planting density. Combining a high density of 25 plants per vessel and a culture medium volume of 40 mL increased the plant height, while the number of shoots per explant was higher with a lower planting density and not influenced by the volume of culture medium [29]. Furthermore, the effect of planting density was assessed under photoautotrophic conditions. Fresh and dry weight, leaf area and number of unfolded leaves of photoautotrophically grown in vitro potato plants were significantly affected by the planting density, and a decrease of CO_2_ concentration, net photosynthetic rate, and mineral concentration was observed with increasing planting density [30]. In another study, in vitro potato plantlets (cv. ‘Benimaru’) decreased their growth with increasing plantlet density when they were incubated under photoautotrophic conditions with a constant number of gas exchanges and a photosynthetic photon flux rate [31]. 

The volume of tissue culture vessels has a major impact on the development and growth of in vitro plants. For example, protocorm-like bodies (PLBs) of *Dendrobium* orchid (cv. ‘Sabin Blue’) showed a 1.5 to 5-times higher growth index in 470 mL vessels compared to smaller vessel volumes of 120, 240, and 350 mL. Interestingly, in plastic vessels of 240 and 350 mL, a higher number of more developed stomatal pores was observed [32]. Likewise, six *Prunus avium* genotypes grown in larger 100 mL Erlenmeyer flasks were shown to have a 1.4 to 2-times higher multiplication rate compared to 30 × 160 mm test tubes [33], whereas in vitro plants of peach (rootstock GF 677) best developed in square vitro vent containers (830 mL) and glass jars (400 mL) in terms of growth homogeneity, multiplication coefficient, and root growth [34].

The objective of the present study was to assess the effect of gas exchange rates (different types of HEPA filter), volume of in vitro containers, and planting density (quantity of plants per area) on growth, development, ion absorption (NO_3_^−^, Ca^++^, K^+^, Na^+^), chlorophyll, and moisture content of in vitro plants of seven potato landraces. To the best knowledge of the authors, it is the first time a study assesses the effect of the combination of these factors on a set of diverse potato genotypes under in vitro conditions.

## 2. Materials and Methods

In vitro plants of seven potato landraces were obtained from the in vitro collection of the CIP genebank (Table 1). Plants were sub-cultured in 25 × 150 mm test tubes on 9 mL of solid MS medium with vitamins [9], placing 4–5 uninodal stem segments per tube. Only the two upper thirds of the stems were used to obtain the stem segments.

The MS medium was supplemented with 25 g L^−1^ sucrose and 6 g L^−1^ of agar. Prior to autoclaving, the pH was adjusted to 5.60 ± 0.02. The plants were incubated at 20 ± 2 °C, with a 16 h/8 h photoperiod, and light intensity of 65 ± 10 μmol m^−2^ s^−1^ (fluorescent tubes, 36 W, cool daylight). The plants were subcultured every 21–24 days until the required plant quantity for setting up the experiment was obtained. 

For the setup of the experiment, uninodal stem segments coming from 21–22 old in vitro potato plants were propagated in special autoclavable plastic vessels of 5 and 12 cm in height (diameter: 9 cm) (SAC O2, Deinze, Belgium) [35] whose lids were equipped with three different types of HEPA filters. The total gas volume contained in the vessel was exchanged 10-, 16-, or 81-times per day, depending on the filter type (based on the vessel model O118/80). Two different vessel volumes, 360 and 870 mL, and three planting densities per vessel, 5 × 5 (25 plants/vessel), 7 × 7 (49 plants/vessel), and 10 × 10 (100 plants/vessel), were assessed for each of the three filter types. Per vessel, 100 mL of filter-sterilized MS medium was dispensed (same composition as above), and plants were incubated for 27–29 days under the same environmental conditions as previously described. Once the incubation period was concluded, 5, 10, or 20 plants were sampled randomly, depending on the total number of plants contained in each vessel (i.e., 25–100 in vitro plants/vessel), and used for sequential measuring of chlorophyll content, biometric data, and ion content in plant sap (Ca^++^-, K^+^-, Na^+^-, and NO_3_^−^-ions). The remaining not-sampled plants were used for the determination of the plants’ moisture content (destructive method).

As control treatments, in vitro potato plants were subcultured in three vessel types routinely used for genebank operations at the CIP: (a) 25 × 150 mm glass test tube (in vitro conservation and propagation: 5 plants/vessel; vessel volume: 50 mL; culture media: 10 mL); (b) 13 × 100 mm glass test tube (international distribution of in vitro plants: 1 plant/vessel; vessel volume: 9 mL; culture media: 2 mL); and (c) GA7^®^ magenta vessel (national and CIP internal distribution: 5 × 5 plants/vessel; vessel volume: 575 mL; dimensions: 77 mm × 77 mm × 97 mm; culture media: 50 mL). The plants of the control treatments were incubated and processed as previously described for the plastic vessels with a HEPA filter. 

Chlorophyll content of leaves (in ppm) was determined in middle-positioned leaves with a hand-held SPAD 502 (Spectrum Technologies, Aurora, IL, USA) chlorophyll meter, suitable for measurements of small leaves. 

**Figure 1:**
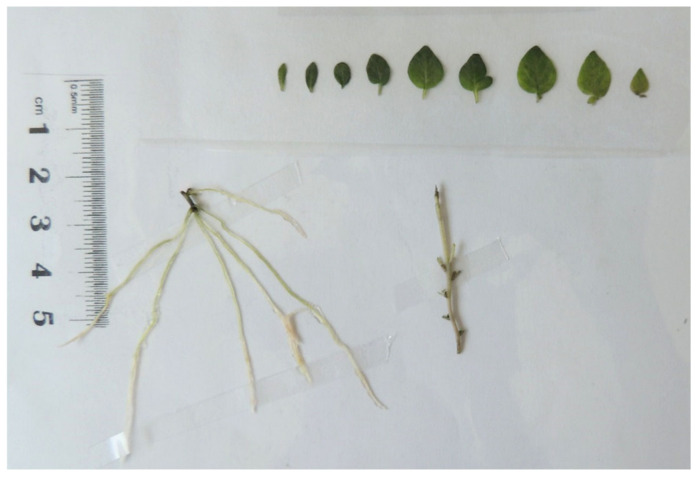
Leaves, stem, and roots of in vitro potato plants mounted on transparent auto-adhesive tape for biometric measurements.

For the measurement of the biometric data, leaves of the sampled plants were removed using a scalpel, laid out onto a transparent auto-adhesive tape (Layconsa, Lima, Peru), and stuck on a white carton evolved in a plastic sheet. Stem and roots were extended and mounted on the sheet using small strips of transparent adhesive tape (Figure 1). The sheets were labeled with accession and treatment identifiers, as well as a software calibration ruler. High resolution photos (4608 × 3456 pixels) of each sheet were taken, and the following biometric variables were measured using ImageJ 1.53t software: stem and root length (cm), number of nodes, stem width in three positions (base, middle, tip) [mm], and individual leaf area (cm^2^). 

To determine the ion-content, sap was extracted using a handheld plant sap press. Employing a micropipette, 200 μL of sap was placed on the flat sensor of each of the pocket hold ion meters Ca-11, K-11, NO3-11, and Na-11 (Horiba, Irvine, CA, USA), to measure the Ca^++^, K^+^, Na^+^, and NO_3_^−^-ion concentration in the plant sap (in ppm), respectively. The ion meters were recalibrated after 6–8 measurements. 

The moisture content (%) of the in vitro potato plants was determined with a MB120 moisture analyzer (Ohaus, Parsippany, NJ, USA). Plant parts were dried at 105 °C for 15–20 min; the equipment automatically performs the measurement of fresh and dry weights (drying cycle without ramp) and calculates the tissue’s moisture content (%). The equipment was auto-calibrated after each processed treatment.

The experiment was set up as a four-factor experiment, with planting density, vessel volume, filter type, and potato genotype as the main factors. Per treatment of each repetition, three vessels were installed and sampled. The experiment was repeated three times in different time periods and examined using the Analysis of Variance method (ANOVA). Prior to ANOVA, data distribution and homogeneity of variance were analyzed, applying the Kolmogorov–Smirnov (*p* > 0.05) and Leven tests (*p* > 0.05), respectively. Logarithmic and Johnson data transformations were applied prior to ANOVA analysis when required. Variables that showed significant differences for ANOVA were differentiated with a multiple comparison test (Tukey; *p* < 0.05). Variables showing strong skewness from normal distribution were analyzed with the Kruskal–Wallis multiple comparison test (*p* < 0.05). Data were analyzed and presented using MINITAB 22.1.0, SPC 6.0.2.1 for Excel, and Python 12.3.2 software.

## 3. Results

**Figure 2:**
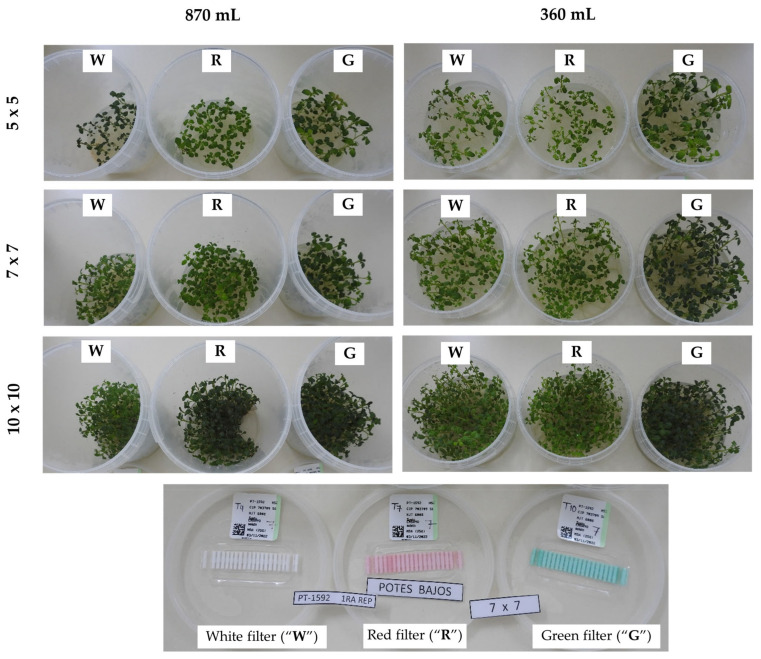
In vitro potato plants grown in plastic culture vessels with different gas exchange rates (green [“G”]: high rate; red [“R”]: medium rate; white [“W”]: low rate), planting densities (5 × 5, 7 × 7, and 10 × 10), and vessel sizes (360 and 870 mL). “POTES BAJOS”: Low vessel (360 mL).

All biometric variables, from stem and root length, number of nodes and stem diameter to total leaf area were very significantly impacted by the genotype, filter type (i.e., gas exchange rate), vessel volume, and plant density (Figure 2).

A higher gas exchange rate (green filter), vessel volume (870 mL), and plant density (10 × 10) significantly increased plant growth in terms of stem length. On the genotype level, CIP 706250 and CIP 704087 developed significantly higher and lower plant heights, respectively, compared to the other five accessions.

The gas exchange rate and vessel volume did not significantly affect root growth. Plants grown with lower density developed a longer maximum root length (5 × 5: 6.0 cm; 7 × 7: 5.8 cm) compared to the high-density planting of 10 × 10 (max. root length of 5.1 cm). CIP 701611, CIP 703709, CIP 705127, and CIP 706250 did develop significantly longer roots than the other three accessions. Although CIP 705127 had the second-lowest stem length, it developed the longest maximum root length (6.3 cm).

While plant density and vessel volume did not influence the number of nodes that were formed, plants grown under a lower gas exchange rate (red and white filter) developed a significantly higher number of nodes (7.5 to 7.6) compared to the filter system with a high gas exchange rate (green filter; 7.1 nodes). CIP 704087 and CIP 701611 formed significantly fewer nodes (6.3 and 6.5) compared to the other five accessions.

Stem width, as a quality criterion for plant robusticity, was notably influenced by gas exchange rate, plant density, and vessel volume. Plants grown under a high gas exchange rate (green filter) developed thicker stems (0.95 mm) compared to lower rates (red and white filter), with stem widths ranging from 0.87 to 0.89 mm. As expected, a high plant density of 10 × 10 plants per vessel resulted in significantly thinner stems (0.83 mm). In a similar way, plants grown at intermediate plant density (7 × 7) had thinner stems of 0.91 mm compared to the low-density pattern of 5 × 5 plants (stem width: 0.97 mm). A higher vessel volume of 870 mL contributed to a significant increase in the stem width (0.92 mm) compared to the smaller vessels of 360 mL (stem width of 0.88 mm). CIP 705127 and CIP 703709 developed significantly thinner stems compared to the other five accessions (Table 2).

**Figure 3:**
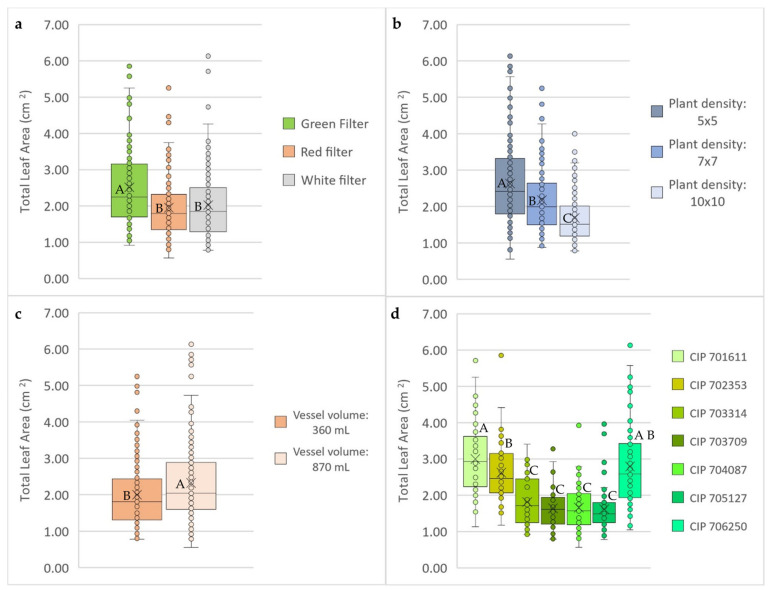
Effect of (**a**) filter type of vessel (green, red, and white), (**b**) plant density (5 × 5, 7 × 7, and 10 × 10), (**c**) vessel volume (360 and 870 mL), and (**d**) genotype (CIP 701611, CIP 702353, CIP 703314, CIP 703709, CIP 704087, CIP 705127, and CIP 706250) on total leaf area (cm^2^) of in vitro potato plants. Mean values and individual data points are shown as crosses and filled circles, respectively. Different capital letters indicate significant differences for Tukey’s multiple comparison test (*p* < 0.05).

A high gas exchange rate (green filter), low plant density (5 × 5), and larger vessel volume (870 mL) positively affected the total leaf area of the plants. Plants grown in vessels with a green filter developed a significantly higher total average leaf area (2.51 cm^2^) compared to the filter systems with lower gas exchange rates (red filter: 1.95 cm^2^; white filter: 2.01 cm^2^). Under a plant density of 5 × 5, the leaf area of the potato in vitro plants increased by a factor of 1.5 (leaf area: 2.63 cm^2^) versus high density planting of 10 × 10 plants per vessel (leaf area: 1.68 cm^2^). A higher vessel volume of 870 mL significantly increased the total average leaf area (2.32 cm^2^) compared to the smaller vessels of 360 mL (2.00 cm^2^). The genotypes CIP 701611, CIP 702353, and CIP 706250 developed significantly larger leaves (2.62 to 3.00 cm^2^) compared to CIP 703314, CIP 703709, CIP 704087, and CIP 705127 (1.62 to 1.81 cm^2^) (Figure 3).

**Figure 4:**
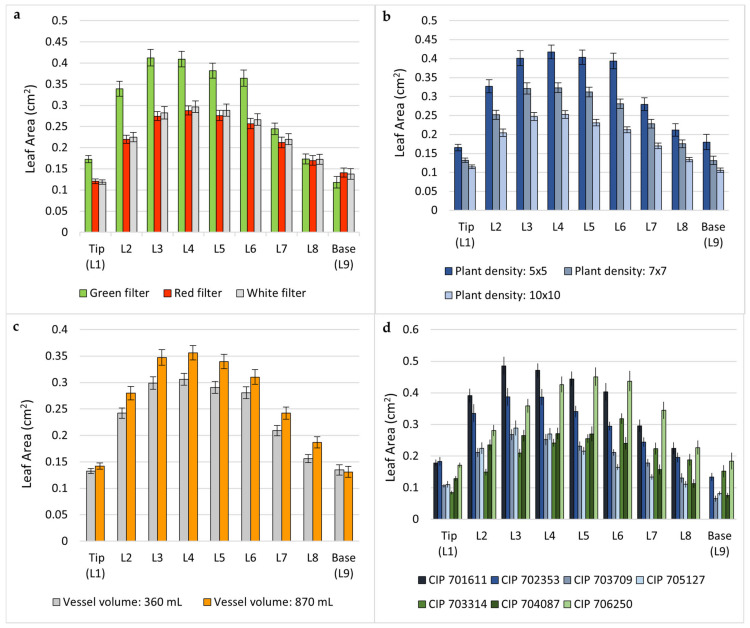
Effect of (**a**) filter type of vessel (green, red, and white), (**b**) plant density (5 × 5, 7 × 7, and 10 × 10), (**c**) vessel volume (360 and 870 mL), and (**d**) genotype (CIP 701611, CIP 702353, CIP 703314, CIP 703709, CIP 704087, CIP 705127, and CIP 706250) on individual leaf size (cm^2^) of in vitro potato plants, depending on the leaf position on the plant (uppermost leaf: L1; lowest leaf: L9). The three genotypes highlighted in green (**d**) showed a different leaf area pattern compared to the rest. Standard errors are shown as bars.

Plotting the area of the individual leaves from up (L1, tip) to down (L9) showed that plants grown under a higher gas exchange rate (green filter) developed larger leaves for leaf positions 1 to 7, but not for the two leaves located at the two lowest positions (L8 and L9). In all positions (L1 to L9), leaves were larger under a low plant density approach (5 × 5) compared to the intermediate (7 × 7) and high plant densities (10 × 10). Also, the intermediate plant density (7 × 7) resulted in larger leaves, from L1 to L9, related to a dense planting of 10 × 10. Likewise, the plants developed bigger leaves in all positions (L1 to L9) when they were grown in larger vessels (870 mL) instead of smaller vessels (360 mL). Four of seven accessions, CIP 701611, CIP 702353, CIP 703709, and CIP 705127 showed a very similar leaf pattern from L1 to L9, with a peak of leaf area at the 3rd-most upper leaf (L3). In contrast, CIP 703314, CIP 704087, and CIP 706250 had their largest individual leaf in position five (L5) or six (L6) [Figure 4].

A very low-to-moderate correlation between the biometric variables of stem and root length, number of nodes, stem diameter, and total leaf area was observed (−0.31 ≤ r ≤ 0.55). It was shown that a higher gas exchange rate (green filter) increased the development of leaf area, stem length, and diameter of the in vitro potato plants but reduced the number of nodes formed. Under a high plant density (10 × 10), stem growth was stimulated, but a decrease in root growth and stem width was observed. In contrast, under a low density of 5 × 5, better root growth and thicker stems were obtained. Plants grown in larger vessels (870 mL) produced longer and thicker stems (Table 2). 

The chlorophyll content in leaves was affected by genotype, gas exchange rate (filter type), and planting density. CIP 703709 and CIP 704087 had a significantly higher average chlorophyll content (32.6–34.0 ppm) in their leaves compared to the other five genotypes (22.0–29.0 ppm). A high gas exchange rate of the vessel (green filter) significantly increased the leaves’ chlorophyll content in all genotypes (32.2 ppm) compared to the lower rates of the red and white filters (25.5 ppm) (Figure S1).

**Figure 5:**
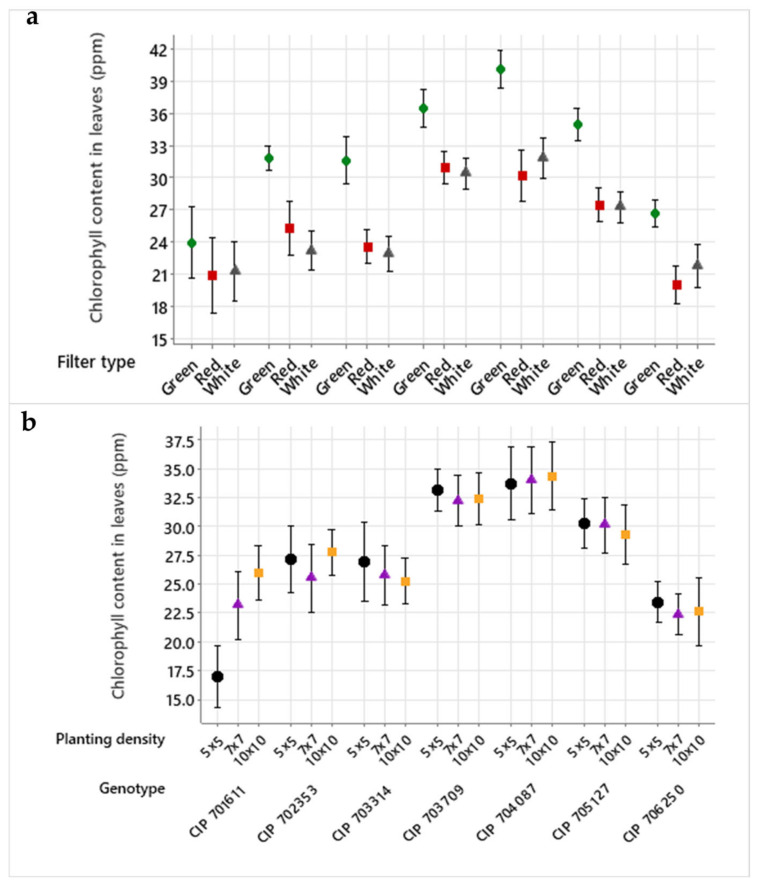
Interaction plots of chlorophyll content in leaves for (**a**) genotype × filter type: data points for the three different filter types are highlighted with circles (green filter), squares (red filter), and triangles (white filter); and (**b**) genotype × planting density: different planting densities are indicated as circles (5 × 5), triangles (7 × 7), and squares (10 × 10). The bars of both graphs show the mean’s confidence level at 95%, using individual standard deviations for interval calculation.

However, the ANOVA analysis also revealed significant differences in the interactions between genotype × filter type and genotype × planting density. While CIP 702353, CIP 703314, and CIP 703709 had a higher chlorophyll content in their leaves with the red filter (intermediate gas exchange rate), CIP 701611, CIP 704087, and CIP 706250 developed a higher content with the white filter (lowest gas exchange rate). Whereas three of seven accessions (CIP 701611, CIP 702353, and CIP 704087) produced a higher chlorophyll content in their leaves under a planting density of 10 × 10, the four other accessions showed the reverse response (CIP 703314, CIP 703709, CIP 705127, and CIP 706250), i.e., the lowest planting density of 5 × 5 led to a higher average chlorophyll content in the plant leaves (Figure 5). 

**Figure 6:**
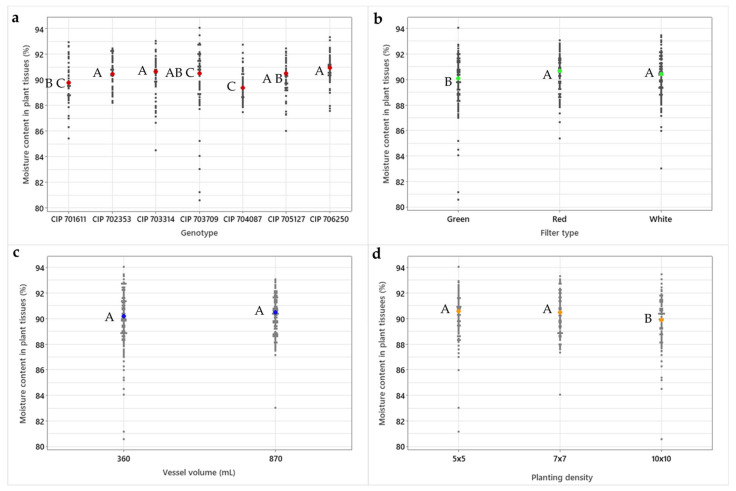
Effect of (**a**) genotype, (**b**) filter type (gas exchange rate), (**c**) vessel volume, and (**d**) planting density on the moisture content in tissues of in vitro potato plants. Individual data points and median values are highlighted as black and colored circles, respectively. Different capital letters indicate significant differences for the Kruskal–Wallis multiple comparison test (*p* < 0.05).

Moisture content in plant tissues was affected by genotype, filter type, and planting density. CIP 706250 had a significantly higher median value of moisture content in its tissues (90.94%) compared to CIP 701611 (89.78%) and CIP 704087 (89.40%). In vitro plants grown under a high gas exchange rate (green filter) presented a significantly lower median value of moisture (90.11%) compared to the intermediate gas exchange rate (red filter; 90.65%). Vessel volume did not affect the moisture content in the plant tissue, in contrast to the planting density. A high planting density of 10 × 10 produced plants with a significantly lower water content (89.9%) compared to the 7 × 7 (90.50%) and 5 × 5 (90.61%) planting schemes (Figure 6).

CIP 701611 and CIP 702353 accumulated significantly higher Ca^++^- (234 and 190 ppm) and Na^+^-ion concentrations (112 and 114 ppm) in their tissues compared to the other five accessions. Versus CIP 704087 (Ca^++^-ion concentration of 51 ppm), CIP 701611 had a 4.6-times higher Ca^++^-ion concentration in its tissues. Inversely, CIP 701611 was characterized by having significant lower NO_3_^−^-(3000 pm) and K^+^-ion concentrations (2724 ppm) in its tissues. Moreover, CIP 702353, although having similar Ca^++^- and Na^+^-ion patterns as CIP 701611, showed the opposite response for the K^+^- and NO_3_^−^-ions compared to CIP 701611. It had the second highest K^+^-ion concentration of all accessions (3407 ppm) and no significant differences in its NO_3_^−^-ion concentration (3672 ppm) compared to the other accessions were observed (Table 3). This might suggest that CIP 701611 and CIP 702353 have different mechanisms for ion-housekeeping and the establishment of osmotic gradients. Nevertheless, a deeper screening with genetically closely related accessions is required before robust conclusions can be drawn. 

The gas exchange rate significantly affected the Ca^++^-ion concentration in plant tissues. With a decreasing gas exchange rate (white filter), the Ca^++^-ion concentration increases (143 ppm), compared to a high gas exchange rate (green filter; 125 ppm). As calcium plays a structural role in cell walls and membranes, a lower availability of O_2_ and CO_2_, or a potential gas accumulation within the vessel, may trigger some kind of protection mechanism. 

Vessel volume did not significantly affect ion concentration within tissues for any of the assessed ions (NO_3_^−^, K^+^, Ca^++^, Na^+^). However, planting density did significantly affect NO_3_^−^, K^+^-, and Na^+^-ion absorption. Plants grown with a low planting density (5 × 5) accumulated a significantly higher concentration of NO_3_^−^ (4003 ppm) and K^+^-ions (3323 ppm) compared to the high-density planting approach of 10 × 10 (NO_3_^−^: 3243 ppm; K^+^: 2896 ppm). In the case of the Na^+^-ion, low-density planting (5 × 5) resulted in a significantly lower Na content (79 ppm) compared to a high-density planting of 10 × 10 (87 ppm) (Table 3).

No correlation was observed between biometric and ion-content variables, but a moderate to high correlation was observed between the concentrations of Ca^++^ vs. Na^+^ (r = 0.503), and NO_3_^−^ vs. K^++^ (r = 0.617), respectively (Figure S2). 

The comparison of the best treatment, i.e., green filter, 5 × 5 planting density, and vessel volume of 870 mL (GF-Control), with the response of in vitro potato plants in routinely used culture vessels during in vitro operations at CIP (13 × 100 mm, 25 × 150 mm test tubes, and GA7^®^ magenta vessels), showed significant differences for multiple variables. The average root length of plants was significantly higher in GF-Control and GA7^®^ vessels (6.2–6.5 cm) compared to plants grown in test tubes (4.3–4.5 cm). No significant differences were observed for the means of plant height between the different vessel types (3.3–3.9 cm). In contrast, in vitro plants developed significantly more robust stems in GF-Control vessels (0.11 cm) compared to the other vessel types (0.09 cm) (Figure S3).

The average number of nodes per plant was very homogenous between vessel types, ranging from 7.2 to 7.4, but significant differences were observed for the variables of total leaf area and chlorophyll content. The in vitro plants developed a significantly higher average total leaf area in GF-Control vessels (3.3 cm^2^) in contrast with the other vessel types (1.9–2.4 cm^2^). Inversely, plants grown in narrow 13 × 100 mm vessels had a significantly higher chlorophyll content (35.8 ppm) in comparison to the GF-Control and GA7^®^ culture vessels (27.9–32.6 ppm). Also, the chlorophyll content of plants grown in GA7^®^ vessels (27.9 ppm) was significantly lower compared to the GF-Control vessels (32.6 ppm) (Figure S4). In vitro potato plants grown in HEPA-filter-equipped vessels with a high gas exchange rate and a low planting density of 5 × 5 (CF-Control) developed significantly thicker stems and a higher total leaf area compared to GA7^®^ culture vessels (Figures S3 and S4). 

## 4. Discussion

In vitro propagation of plants forms part of experiments, operational genebank activities, and commercial applications. Nevertheless, only a few and limited studies were performed on the effect of the culture vessels’ gas exchange rate and volume, along with the assessment of different planting densities and genotypes. In the present study, it was confirmed, in accordance with previous reports [17,18,19,20,21], that plant growth (stem height) and photosynthetic activity (chlorophyll content) of in vitro potato plants are stimulated when they are subcultured in culture vessels with a high gas exchange rate.

A 26% higher chlorophyll content was observed for in vitro potato plants cultured under a regime of a high gas exchange rate (green filter) compared to the lowest assessed rate (white filter). Although this increase is lower compared to what was previously reported by Genoud-Gourichon et al. [18], the sealing and vessel types varied significantly between these two studies. As they used completely sealed test tubes as a control treatment, the observed 70% difference in photosynthetic activity between vented and sealed vessels could be a consequence of a total inhibition of gas exchange or an excessive accumulation of ethylene, as reported by Zobayed et al. [20].

Beyond this and supported by the findings from Habibi and Purohit and Askari et al. [23,24], in vitro potato plants may be able to switch to alternative or complementary autotrophic behaviors, although they are grown under heterotrophic conditions on sucrose-rich culture medium. 

Regarding the effect of high planting densities, previous studies reported contradictory results. While Shakeel et al. [27] observed an increase in the number of nodes of in vitro potato plants when grown under a higher planting density (five plants per tube), Sarkar et al. [28] did not report significant differences for the same variable. In the current study, planting density had no effect on the number of nodes that the plants developed, but under a high density of 100 plants per vessel (10 × 10), an increase of 24% in plant height was observed, along with a reduction in stem diameter (17%), compared to a lower 25-plant density (5 × 5). Nevertheless, under a high planting density, it was possible to increase the multiplication rate per culture vessel by a factor of four (25 vs. 100 plants per vessel), resulting in an economical savings in terms of personnel and supply costs.

Plants grown under a high planting density (10 × 10) had a higher concentration of Na^+^-ions in their tissue, along with a decrease in K^+^-ions, compared to plants grown under a low planting density (5 × 5). It is suspected that under high planting densities, the K^+^-uptake mechanism of in vitro potato plants is altered, resulting in the activation of a compensation mechanism to increase the uptake of Na^+^-ions. As the cytosolic K^+^/Na^+^-ion ratio is considered one of the best indicators for salt and drought stress [36,37], and the K^+^ concentration in the plant tissue dropped down by 12% under a high planting density (10 × 10), together with a significant decrease in moisture content (Figure 6d), it is suspected that the potato in vitro plants are suffering stress at this planting density (10 × 10).

Moreover, our study showed that a low gas exchange rate stimulated the uptake and/or metabolization of Ca^++^-ions, resulting in a 12% increase of Ca^++^-ions in plant tissue compared to in vitro plants grown under a high gas exchange rate. Ca^2+^ has a key function in multiple metabolic pathways related to plant growth and development, stress resistance, hormonal response, and photosynthesis [38]. In more fundamental studies, it has been shown that Ca^2+^ can affect the gas exchange of photosynthesis by controlling the opening and closure of the stomas [39,40]. Hence, it could be possible that potato in vitro plants absorb more Ca^2+^ under reduced CO_2_ availability (low gas exchange rate) in order to readjust the stomata control. Nevertheless, to confirm this supposition, it is necessary to perform additional fundamental studies using proteomics and transcriptomics, along with real-time environmental data measurements within the vessel (CO_2_, O_2_, ethylene, temperature, relative humidity, etc.).

In the present study, the size of the in vitro culture vessel (volume) significantly affected stem length and width, total leaf area, and chlorophyll content of the in vitro plants, but had no impact on ion concentration (K^+^, Na^+^, Ca^++^, NO_3_^−^) or moisture content. A similar tendency to increase growth was observed in other studies when in vitro plants were subcultured in larger vessels [32,33,34]. In our study, increasing the vessel volume by a factor of 2.4 (360 mL vs. 870 mL) resulted in increases of 12%, 32%, and 4% for plant height, total leaf area, and chlorophyll content, respectively. The reduction in chlorophyll content in smaller vessels (360 mL) could be the direct consequence of a reduced total leaf area. 

As the seven potato cultivars only exceptionally showed significant differences (Figure 5) for the interaction of the principal factors of gas exchange rate, planting density, and vessel volume, and maintained a homologous response tendency within each of the genotypes, it is assumed that space (plant density and vessel volume) and availability, accumulation, and/or exchange of gases cause a general effect on the plant physiology of potato plants grown under in vitro conditions. The best results in terms of plant quality were observed when the in vitro potato plants were grown at a high gas exchange rate (green filter) and low planting density (5 × 5) in larger-sized in vitro culture vessels (870 mL).

Under these conditions, the plants of the control treatments, i.e., GA7^®^ magenta vessels, 13 × 100 mm, and 25 × 150 mm glass test tubes, developed weaker plants in terms of stem width and total leaf area (Figures S3 and S4). As the here assessed vessel types are designed for mass propagation of in vitro plants, especially the comparison with the GA7^®^ magenta control treatment is important. Because the HEPA-filter-equipped in vitro culture vessels showed a superior response compared to GA7^®^ magentas, along with a lower vessel cost, CIP is planning to use these kinds of vessels for the distribution of germplasm on an institutional and national level.

Our study concludes that the development and quality of in vitro potato plants are greatly enhanced by adjusting in vitro settings, particularly gas exchange rate, planting density, and vessel volume. Elevated gas exchange rates improve plant height, leaf area, and chlorophyll content, while high planting densities can cause stress to plants due to changes in ion uptake. Better overall growth metrics are produced by larger vessel volumes. Additionally, the study emphasizes the benefits of HEPA-filter-equipped vessels over conventional GA7^®^ magenta vessels for germplasm dissemination and propagation. To improve in vitro propagation methods, future research examining these characteristics across various plant species is warranted.

## Figures and Tables

**Table 1:** Description of seven potato accessions used as plant material in the study. STN = *S. stenotomum* subsp. *stenotomum*; GON = *S. stenotomum* subsp. *goniocalyx*; ADG = *S. tuberosum* subsp. *andigena*.

Accession Identifier	GLIS-DOIs	Species/Subspecies	Ploidy Level	Country of Origin		Collection Site		
						Elevation (MASL)	Latitude	Longitude
CIP 701611	10.18730/96W1	GON	2x	Peru		3425	−11.6781	−75.0946
CIP 702353	10.18730/9CM0	STN	2x	Peru		2923	−13.3185	−71.5955
CIP 703314	10.18730/9KYC	STN	2x	Peru		3394	−14.4547	−69.5371
CIP 703709	10.18730/9XRY	STN	2x	Peru		4096	−11.0879	−76.1475
CIP 704087	10.18730/A8A=	ADG	4x	Bolivia		3362	−19.8936	−65.4822
CIP 705127	10.18730/B5RF	ADG	4x	Colombia		2634	5.15	−73.6833
CIP 706250	10.18730/C5G*	STN	2x	Bolivia		3420	−17.4333	−65.7166

**Table 2:** Effect of genotype, gas exchange rate (filter type), plant density, and vessel volume on biometric variables of stem and root length, number of nodes, and stem diameter of potato in vitro plants. Different capital letters indicate significant differences for Tukey’s multiple comparison test (*p* < 0.05) within each of the principal factors. SE: Standard Error.

Factors		Biometric Variables (*)														
		Stem Length (cm)	±	SE		Root Length (cm)	±	SE		Number of Nodes	±	SE		Stem Diameter (mm)	±	SE
**Genotype**																
CIP 701611		3.43 ^B^	±	0.17		5.57 ^A B C^	±	0.16		6.5 ^C^	±	0.1		1.06 ^A^	±	0.04
CIP 702353		3.25 ^B^	±	0.07		5.10 ^C^	±	0.15		8.1 ^A^	±	0.1		0.86 ^B^	±	0.03
CIP 703314		2.92 ^C^	±	0.08		5.04 ^C^	±	0.23		7.7 ^A B^	±	0.2		0.97 ^A^	±	0.03
CIP 703709		3.07 ^B^	±	0.10		6.16 ^A B^	±	0.14		7.5 ^B^	±	0.1		0.72 ^C^	±	0.01
CIP 704087		2.09 ^D^	±	0.12		5.49 ^B C^	±	0.19		6.3 ^C^	±	0.1		1.00 ^A^	±	0.03
CIP 705127		2.98 ^C^	±	0.09		6.25 ^A^	±	0.16		8.1 ^A^	±	0.1		0.67 ^C^	±	0.01
CIP 706250		3.86 ^A^	±	0.11		5.74 ^A B C^	±	0.20		7.6 ^A B^	±	0.1		1.03 ^A^	±	0.03
**Filter type**																
Green		3.26 ^A^	±	0.08		5.73 ^A^	±	0.11		7.1 ^B^	±	0.1		0.95 ^A^	±	0.02
Red		2.98 ^B^	±	0.09		5.51 ^A^	±	0.13		7.5 ^A^	±	0.1		0.89 ^B^	±	0.02
White		3.01 ^B^	±	0.08		5.63 ^A^	±	0.13		7.6 ^A^	±	0.1		0.87 ^B^	±	0.02
**Plant density**																
5 × 5		2.76 ^C^	±	0.09		6.04 ^A^	±	0.14		7.4 ^A^	±	0.1		0.97 ^A^	±	0.02
7 × 7		3.08 ^B^	±	0.08		5.75 ^A^	±	0.11		7.5 ^A^	±	0.1		0.91 ^B^	±	0.02
10 × 10		3.42 ^A^	±	0.07		5.08 ^B^	±	0.10		7.3 ^A^	±	0.1		0.83 ^C^	±	0.02
**Vessel volume**																
360 mL		2.91 ^B^	±	0.07		5.60 ^A^	±	0.10		7.3 ^A^	±	0.1		0.88 ^B^	±	0.02
870 mL		3.27 ^A^	±	0.07		5.65 ^A^	±	0.10		7.4 ^A^	±	0.1		0.92 ^A^	±	0.02

(*) Observations: Data on stem and root length and number of nodes have a normal or close to normal distribution, with *p* values ranging from 0.04 to >0.13 (Kolmogorov–Smirnov test [K–S test]). No data transformation was required. Data of stem diameter were transformed with the Johnson transformation (*p* = 0.10) prior to the ANOVA analysis. The *p*-values of the K–S test were >0.01 and 0.71 before and after data transformation, respectively. Stem width measurements were taken at the base, center, and tip sections of the stem and averaged for data analysis.

**Table 3:** Effect of genotype, gas exchange rate (filter type), plant density, and vessel volume on ion concentration of NO_3_^−^, K^+^, Ca^++^, and Na^+^ in plant sap of potato in vitro plants. Different capital letters indicate significant differences for Tukey’s multiple comparison test (*p* < 0.05) within each of the principal factors. SE: Standard Error.

Factors		Ion-Content in Plant Sap (in ppm)														
		NO_3_^−^ (*)	±	SE		K^+^	±	SE		Ca^++^	±	SE		Na^+^	±	SE
**Genotype**																
CIP 701611		3000 ^B^	±	137		2724 ^C^	±	155		234 ^A^	±	12		112 ^A^	±	5
CIP 702353		3672 ^AB^	±	250		3407 ^A^	±	103		190 ^A^	±	8		114 ^A^	±	4
CIP 703314		3881 ^A^	±	143		3211 ^A B C^	±	132		110 ^B C^	±	5		72 ^B^	±	1
CIP 703709		3756 ^A^	±	123		2900 ^B C^	±	71		137 ^B^	±	6		66 ^B^	±	1
CIP 704087		4339 ^A^	±	235		3541 ^A B^	±	152		51 ^D^	±	3		76 ^B^	±	2
CIP 705127		3756 ^A^	±	152		3002 ^A B C^	±	96		102 ^C^	±	4		69 ^B^	±	2
CIP 706250		3739 ^A^	±	112		3268 ^A B^	±	99		115 ^B C^	±	5		71 ^B^	±	3
**Filter type**																
Green		3550 ^A^	±	108		3060 ^A^	±	80		125 ^B^	±	7		80 ^A^	±	2
Red		3805 ^A^	±	124		3150 ^A^	±	80		135 ^A B^	±	6		83 ^A^	±	2
White		3848 ^A^	±	117		3242 ^A^	±	83		143 ^A^	±	7		85 ^A^	±	3
**Plant density**																
5 × 5		4003 ^A^	±	110		3323 ^A^	±	80		121 ^A^	±	6		79 ^B^	±	3
7 × 7		3957 ^A^	±	123		3232 ^A^	±	79		134 ^A^	±	6		83 ^A B^	±	2
10 × 10		3243 ^B^	±	105		2896 ^B^	±	80		147 ^A^	±	8		87 ^A^	±	2
**Vessel volume**																
360 mL		3918 ^A^	±	113		3331 ^A^	±	69		135 ^A^	±	5		83 ^A^	±	2
870 mL		3551 ^A^	±	72		2970 ^A^	±	60		134 ^A^	±	6		83 ^A^	±	2

(*) Observation: Data of NO_3_^−^ content in plant sap was analyzed with the Kruskal–Wallis multiple comparison test (non-parametric; α = 0.05), as skewness from normal distribution was still high after data transformation.

## Data Availability

The original data presented in the study are openly available in CIP’s dataverse depository at https://data.cipotato.org/dataverse.xhtml (accesed on 6 October 2024).

## Supplementary Materials

The following supporting information can be downloaded at: https://www.mdpi.com/article/10.3390/plants13192830/s1, Figure S1. Effect of genotype (a), filter type (gas exchange rate) (b), vessel volume (c), and planting density, (d) on the chlorophyl content in leaves of in vitro potato plants; Figure S2. Correlation matrix of NO^3−^, K^+^, Ca^++^, and Na^+^-ions in plant tissues of potato in vitro plants; Figure S3. Violin plots for (a) root length, (b) plant height, and (c) stem diameter (cm) of in vitro plants of seven potato accessions; Figure S4. Violin plots for (a) number of nodes, (b) total leaf area (cm2) and (c) chlorophyll content (ppm) of in vitro plants of seven potato accessions.
