# Supplementary material for: Effect of Gas Exchange Rate, Vessel Type, Planting Density, and Genotype on Growth, Photosynthetic Activity, and Ion Uptake of In Vitro Potato Plants
Source: Plants (Basel). 2024 Oct 9;13(19):2830. doi: 10.3390/plants13192830 (PMC11479155; doi:10.3390/plants13192830)

Figure S1. Effect of genotype (a), filter type (gas exchange rate) [b], vessel volume (c), and planting density, (d) on the chlorophyl content in leaves of *in vitro* potato plants. Individual data points and mean values are highlighted as grey and colored circles, respectively. The bars show the mean's confidence level at 95%, using individual standard deviations for interval calculation. Different capital letters indicate significant differences for Tukey's multiple comparison test ($p<0.05$).

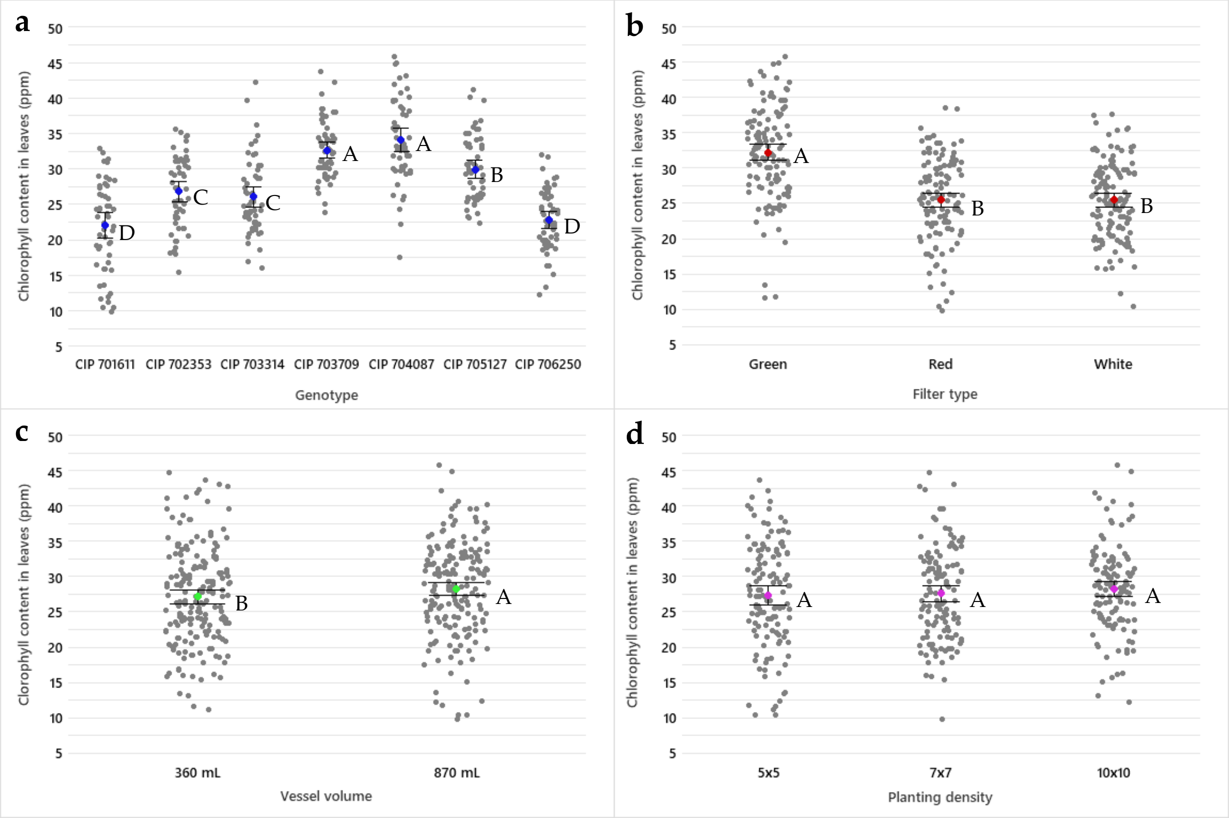

Figure S2. Correlation matrix of NO_3^-, K^+, Ca^{++}, and Na^+-ions in plant tissues of potato *in vitro* plants. The matrix shows the Pearson correlation coefficient (r) for a confidence interval of 95%. Variable correlations with moderate (Ca^{++} vs. Na^+) to high (K^+ vs. NO_3^-) relationship levels were highlighted with a red square.

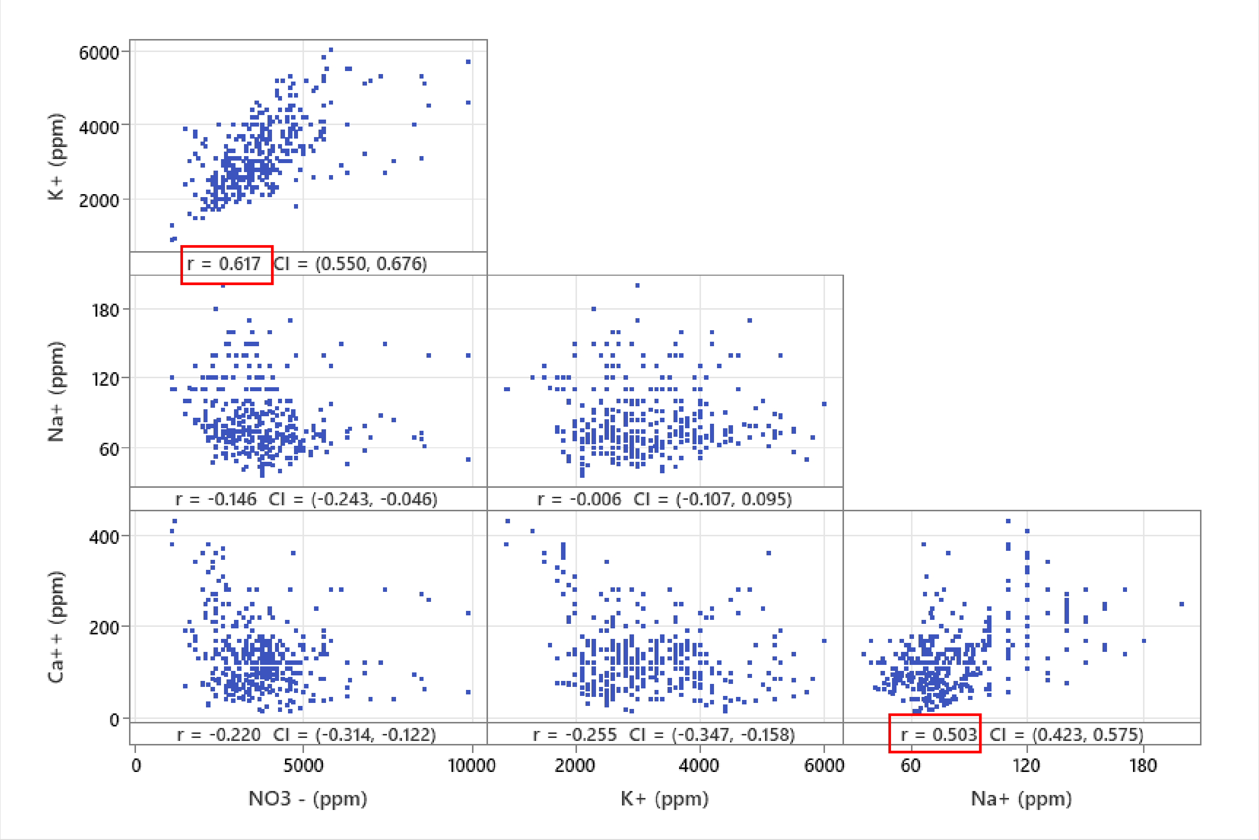

Figure S3. Violin plots for (a) root length, (b) plant height, and (c) stem diameter (cm) of *in vitro* plants of seven potato accessions. The graph shows the results of the best treatment (green filter, 5x5 planting density, 870 mL vessel volume) compared to plants grown in vessels for CIP routine operations (GA7 magenta vessel, 25x150 mm and 13x100 mm test tubes). The mean and median values are shown as a red circle and a white line, respectively. Different capital letters indicate significant differences for Tukey's multiple comparison test ($p<0.05$).

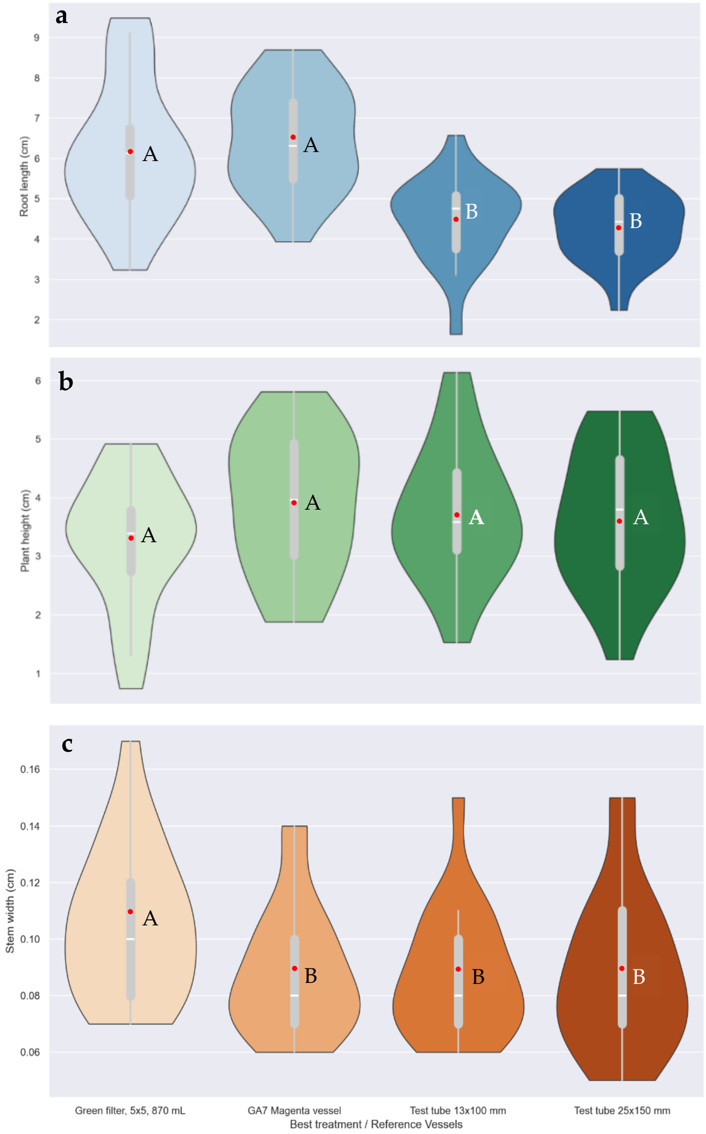

Figure S4. Violin plots for (a) number of nodes, (b) total leaf area (cm^2) and (c) chlorophyll content (ppm) of *in vitro* plants of seven potato accessions. The graph shows the results of the best treatment (green filter, 5x5 planting density, 870 mL vessel volume) compared to plants grown in vessels for CIP routine operations (GA7 magenta vessel, 25x150 mm and 13x100 mm test tubes). The mean and median values are shown as a red circle and a white line, respectively. Different capital letters indicate significant differences for Tukey's multiple comparison test ($p<0.05$).

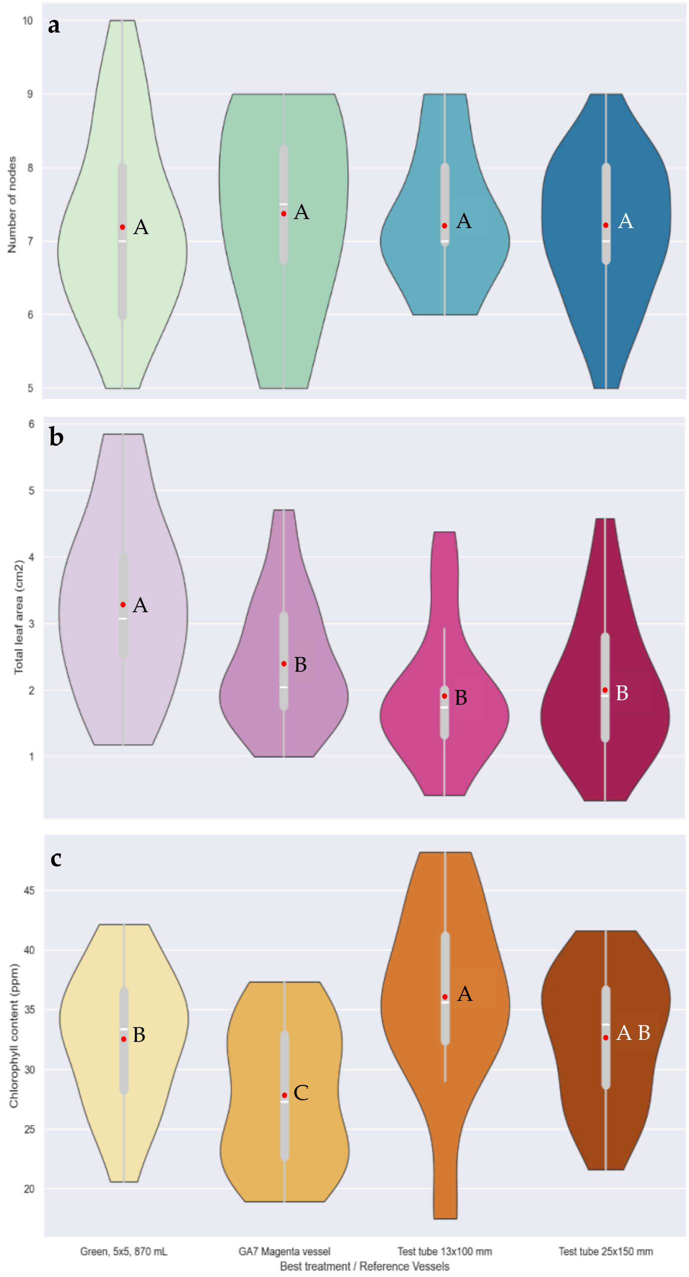

Supplement: Supplementary file 1 [file plants-13-02830-s001.zip › plants-3098243-supplementary.pdf]
